# Supplementary material for: Assessing and visualizing fragility of clinical results with binary outcomes in R using the fragility package
Source: PLoS One. 2022 Jun 1;17(6):e0268754. doi: 10.1371/journal.pone.0268754 (PMC9159630; doi:10.1371/journal.pone.0268754)
Supplement: S1 File — (PDF) [file pone.0268754.s001.pdf]

Additional information of functions' arguments

Appendix A. Additional information of functions for assessing the fragility

For the function `frag.study()`, recall that its arguments include:

```
frag.study(e0, n0, e1, n1, data, all = FALSE, methods,
  modify0 = "both", modify1 = "both", alpha = 0.05,
  alternative = "two.sided", OR = 1, RR = 1, RD = 0, allcase = TRUE)
```

In addition to the arguments detailed in the main content, the argument `methods` specifies the statistical method(s) used to calculate p values and thus determine the significance or non-significance. Five methods mentioned in the main content are available, i.e., Fisher's exact test (`"Fisher"`), the chi-squared test (`"chisq"`), OR (`"OR"`), RR (`"RR"`), and RD (`"RD"`). This argument could include a single method (by specifying a single character string) or multiple methods (by specifying a vector of character strings); its default includes all five possible methods. The two arguments `modify0` and `modify1` dictate how event status is modified in groups 0 and 1, respectively; each argument could be one of `"increase"` (increasing event counts), `"decrease"` (decreasing event counts), `"both"` (the default, modifying event status in both directions), and `"none"` (no modification). In practice, the modifications in the two groups may be determined based on clinicians' opinions. The significance level is given by the argument `alpha` with the default 0.05. The argument `alternative` specifies whether one-sided (`"one.sided"`) or two-sided (`"two.sided"`) p values are produced when using the OR, RR, and/or RD. The p values are always two-sided for Fisher's exact test and the chi-squared test (even if `alternative = "one.sided"`) because they test for the association with no specific direction. One may specify the values of the OR, RR, and RD under the null hypothesis (if the argument `methods` includes some of them) using the arguments `OR`, `RR`, and `RD`. Finally, the logical argument `allcase` indicates whether users would like to obtain all cases of minimal event status modifications for altering significance or non-significance. The default is `TRUE`, and users may change it to `FALSE` to save some computation time if they only need the numerical value of the FI or FQ.

For the function `frag.ma()`, recall that its arguments are:

```
frag.ma(e0, n0, e1, n1, data, measure = "OR", alpha = 0.05,
  mod.dir = "both", OR = 1, RR = 1, RD = 0, method = "DL", test = "z",
  ...)
```

In addition to the arguments detailed in the main content, `method` and `test` are two important arguments for performing the MA; they are passed to `rma.uni()` in "metafor." The `method` specifies the MA method, including the commonly used fixed-effect model (`"FE"`), DL method (`"DL"`), maximum likelihood method (`"ML"`), REML method (`"REML"`), and many others. Of note, the DL method is very popular, but it has been found to be inferior to the REML method [1]; therefore, "metafor" uses the REML method as the default. However, the estimation process may not converge when implementing the REML method, e.g., for some MAs with few studies. As many

MAs with different event status modifications need to be performed to derive the fragility measure, the REML method might lead to computation errors during this process. Therefore, `frag.ma()` uses the DL method as the default, which is based on the method of moments and thus generally does not lead to computational errors. Users should also carefully note that the argument `method` in `frag.ma()` differs from `methods` in `frag.study()`; the latter specifies the method(s) for producing p values of individual studies. Moreover, the argument `test` in `frag.ma()` indicates how CIs of MAs are derived; four options are available, i.e., `"z"`, `"t"`, `"knha"` (the HKSJ method), and `"adhoc"`. The first option indicates Wald-type CIs based on the standard normal distribution (the default), while the latter three yield CIs based on the t distribution. Users may refer to the manual of "metafor" for more details [2]. Most existing MAs use Wald-type CIs based on the standard normal distribution, but recently the HKSJ method is recommended because it generally leads to better coverage probabilities [3]. Finally, many additional arguments from "metafor" can be specified for `frag.ma()`. For example, the arguments `add` and `drop00` may be used for handling studies with zero event counts (i.e., the value of continuity correction and whether double-zero-event studies are removed from the MA).

Appendix B. Additional information of functions for visualizing the fragility

For the object of class `"frag.study.all"`, recall that the arguments of the visualization function are as follows:

```
plot(x, method, modify0, modify1, trun, xlab, ylab, xlim, ylim,
  cex.pts, cex.legend.pval, cex.legend.title,
  col.ori, col.ori.hl, col.f.hl, col.sig, lty.ori, lwd.ori,
  pch, pch.ori, pch.ori.hl, pch.f, pch.f.hl, pch.trun,
  adjust.legend, adjust.seg, legend.pvals, ...)
```

In addition to the arguments detailed in the main content, the argument `trun` specifies the truncation of p values (on a base-10 logarithmic scale); p values smaller than the threshold (i.e., `10^-trun`) are truncated. This helps avoid wide plot ranges caused by extremely small p values. The arguments `xlab`, `ylab`, `xlim`, and `ylim` have the same usage as in the default plot function `plot.default()`. The arguments starting with `cex`, `col`, `lty`, `lwd`, and `pch` are used for specifying the sizes, colors, line types, line widths, and point shapes of certain plot features; see details in the manual of "fragility." The last three arguments adjust the display of the legend when both `modify0` and `modify1` are `TRUE`. Users may specify additional arguments from `plot.default()` to adjust many other graphical parameters.

For an object `x` of class `"frag.alpha"` produced by `frag.study.alpha()`, `frag.ma.alpha()`, or `frag.nma.alpha()`, recall that `plot()` via the S3 method for this class has the following arguments:

```
plot(x, method, fragility = "FI", percentage = TRUE, xlab, ylab,
  xlim, ylim, cex.pts, col.line, col.pval, col.sig, lty.pval,
  lwd, lwd.pval, pch, pch.inf, tid1, tid2, FQ.nma = FALSE, ...)
```

The argument `method` is only used when `x` is also of class `"frag.study.alpha"` produced by `frag.study.alpha()`; if not specified, the first method stored in `x$methods` will be used. Recall that only one effect measure can be specified when using `frag.ma.alpha()` and `frag.nma.alpha()`, so users do not need to specify this argument if `x` is produced by these two functions. The argument `fragility` is either

"FI" (the default) or "FQ", indicating which fragility measure is visualized. When plotting FQs (`fragility = "FQ"`), the argument `percentage` determines whether presenting them in percentage (`TRUE`, the default) or not (`FALSE`). If `x` is of class `"frag.nma.alpha"` produced by `frag.nma.alpha()` for an NMA, users may use the arguments `tid1` and `tid2` to specify the treatment comparison of interest for visualization. The first comparison stored in `x$tid` is used if they are not specified. As mentioned in the main content, two possible types of FQ may be used for an NMA; the logical argument `FQ.nma` determines the type to be plotted. If it is `FALSE` (the default), the FQ is derived with respect to the total sample size of the corresponding treatment comparison; if `TRUE`, the FQ is based on the total sample size among the whole NMA.

For an object of class `"frag.multi"`, recall that the visualization function has the following arguments:

```
plot(x, method, dir = "both", fragility = "FI", percentage = TRUE,
  max.f = NULL, bar, names.arg, space = 0, breaks, freq,
  reverse = FALSE, xlab, ylab, main = NULL, cex.marker, col.border,
  col.sig, trun.marker = TRUE, ...)
```

where `x` is the output from `frag.studies()` and `frag.mas()`. This function generates a bar plot or histogram (depending on the specified arguments) to show the overall distribution of fragility measures among the multiple datasets of individual studies or pairwise MAs. In the bar plot, the x axis presents the values of FIs, and the y axis presents the corresponding frequencies (counts). In the histogram, the x axis presents the intervals of FIs or FQs, and the y axis presents the corresponding frequencies or densities. The argument `method` is only used when `x` is also of class `"frag.studies"` produced by the `frag.studies()` function; it specifies the method for calculating *p* values of individual studies. The argument `dir` specifies the type of fragility measures with a certain direction of significance change to be visualized. The fragility measures of all datasets can be classified into two types, i.e., significance altered to non-significance (`"sig2nonsig"`) and non-significance altered to significance (`"nonsig2sig"`). The argument `dir` can be one of `"sig2nonsig"`, `"nonsig2sig"`, and `"both"` (both directions, the default). If `dir = "both"`, users can use the logical argument `reverse` to change how the two types of fragility measures are displayed (i.e., at the bottom or top) in the plot. The arguments `fragility` and `percentage` specify the fragility measures (FIs or FQs) to be plotted and whether FQs are presented in percentage. Some datasets may have extreme values of their fragility measures; users may use the argument `max.f` to indicate the maximum value to be presented in the plot, so that fragility measures larger than this threshold will be truncated. The default is `NULL`, i.e., no truncation. The logical argument `bar` specifies whether a bar plot (`TRUE`) or histogram (`FALSE`) is generated. The bar plot is only available for FIs (`fragility = "FI"`), which take positive integers; the default is `bar = TRUE` in this case; for FQs (`fragility = "FQ"`), `bar` is always `FALSE`. The arguments `names.arg` and `space` are only used in the bar plot; they specify names to be plotted below each bar and the amount of space between bars, which are passed to `barplot()` in "graphics." Moreover, the logical argument `trun.marker` specifies whether a text, which gives information about the truncation, is displayed at the place of the truncated fragility measures in the histogram (`bar = FALSE`). The arguments `breaks` and `freq` are only used in the histogram; they specify the breaks on the x axis and whether the y axis presents frequencies (`freq = TRUE`) or densities (`freq = FALSE`), which are passed to `hist()` in "graphics." Finally, the remaining arguments are used to set many other graphical parameters, and users can specify additional arguments from `barplot()` (when `bar = TRUE`) or `hist()` (when `bar = FALSE`) to customize the plot.

References

1. Langan D, Higgins JPT, Jackson D, Bowden J, Veroniki AA, Kontopantelis E, et al. A comparison of heterogeneity variance estimators in simulated random-effects meta-analyses. Research Synthesis Methods. 2019;10(1):83–98.

2. Viechtbauer W. metafor: Meta-Analysis Package for R; 2020. Available from: `https://CRAN.R-project.org/package=metafor`.

3. IntHout J, Ioannidis JPA, Borm GF. The Hartung-Knapp-Sidik-Jonkman method for random effects meta-analysis is straightforward and considerably outperforms the standard DerSimonian-Laird method. BMC Medical Research Methodology. 2014;14(1):25.
